# Quantification of rehabilitation for patients with Parkinson’s disease and related disorders using wearable devices; a proof of concept

**DOI:** 10.3389/fneur.2025.1609823

**Published:** 2025-07-23

**Authors:** Hikaru Kamo, Genko Oyama, Koichi Nagaki, Nobutaka Hattori

**Affiliations:** ^1^Department of Neurology, Juntendo University School of Medicine, Bunkyo, Japan; ^2^Department of Neurology, Fixel Institute for Neurological Diseases, University of Florida, Gainesville, FL, United States; ^3^Department of Neurodegenerative and Demented Disorders, Juntendo University Graduate School of Medicine, Bunkyo, Japan; ^4^Department of Home Medical Care System, Based on Information and Communication Technology, Juntendo University Graduate School of Medicine, Bunkyo, Japan; ^5^Department of Drug Development for Parkinson's Disease, Juntendo University Graduate School of Medicine, Bunkyo, Japan; ^6^Department of PRO-Based Integrated Data Analysis in Neurological Disorders, Juntendo University Graduate School of Medicine, Bunkyo, Japan; ^7^Research and Therapeutics for Movement Disorders Juntendo University Graduate School of Medicine, Bunkyo, Japan; ^8^Department of Service Development, Sunwels Co., Ltd., Minato, Japan; ^9^Department of Health Economics, Center for Gerontology and Social Science, Research Institute, National Center for Geriatrics and Gerontology, Obu, Japan; ^10^Research Institute of Disease of Old Age, Graduate School of Medicine, Juntendo University, Bunkyo, Japan; ^11^Neurodegenerative Disorders Collaborative Laboratory, RIKEN Center for Brain Science, Saitama, Japan; ^12^Department of Psychiatry, Juntendo University School of Medicine, Bunkyo, Japan; ^13^Department of Neurosurgery, Juntendo University School of Medicine, Bunkyo, Japan

**Keywords:** Parkinson’s disease, rehabilitation, exercise therapy, wearable device, Parkinson plus syndrome, progressive supranuclear palsy, Corticobasal degeneration, digital biomarker

## Abstract

**Introduction:**

To date, disease-modifying therapies have not been established for Parkinson’s disease (PD) or atypical parkinsonisms. Exercise has been reported to help maintain functional abilities and delay disease progression. However, no consensus exists regarding the type, amount, or timing of exercise for each disease stage. Therefore, this study aimed to quantify rehabilitation interventions and identify optimal approaches based on patient characteristics.

**Methods:**

Participants diagnosed with PD or related disorders and residing in an elderly care facility received various rehabilitation therapies—including water stimulation bed, therapeutic massage, sit-to-stand training, gait training, cycle ergometer training, aerobic training using treadmill, stretching, balance training, calisthenics, and resistance training—while wearing a wearable device between April and May 2022. The following parameters were evaluated: differences in body surface temperature and pulse rate before and after rehabilitation, calories burned, steps taken during rehabilitation, and upper and lower extremity activity indices.

**Results:**

A total of 49 participants were included, and the frequency of rehabilitation sessions was determined at the discretion of the therapist based on each participant’s condition. Each rehabilitation session was quantified and visualized using radar charts.

**Discussion:**

This study offers insight into the quantification and visualization of rehabilitation effects in patients with progressive neurodegenerative diseases presenting with parkinsonism. Future efforts should focus on accumulating data under standardized conditions and assessing motor symptoms longitudinally to develop personalized rehabilitation programs for patients with neurodegenerative disorders.

## Introduction

Parkinson’s disease (PD) and related disorders, including progressive supranuclear palsy (PSP), multiple system atrophy (MSA), and Corticobasal Syndrome (CBS), are common neurodegenerative diseases (1). Although dopamine replacement and certain surgical therapies are established treatment options, their effectiveness is limited, particularly for atypical parkinsonisms (2–4). Furthermore, no disease-modifying therapy has been established to date (5). Exercise has been reported to be not only symptomatically effective but also neuroprotective, potentially reducing the risk of developing neurodegenerative diseases, including PD (6). Consequently, early rehabilitation intervention is recommended in multiple guidelines for patients with PD (7). Although the benefits of rehabilitation and exercise therapy for PD have been reported, evidence for Parkinson plus syndromes remains limited. Additionally, no consensus on the optimal timing, frequency, or intensity of exercise across disease stage (8–10). To address these gaps, quantification of rehabilitation protocols is essential. Most current rehabilitation practices are based on therapist experience, leading to variability in outcomes even when similar interventions are provided for equal durations. Thus, we aimed to quantify individual rehabilitation sessions and explore optimal approaches tailored to patient characteristics.

Recent advances in wearable technologies have enabled objective and quantitative monitoring of motor symptoms in PD and related disorders, offering real-time feedback and enhancing both in-clinic and home-based rehabilitation. A growing body of research has applied wearable sensors—such as inertial measurement units, electromyography, and deformation sensors—for monitoring gait, upper-limb function, and postural control (11). Studies using these systems have demonstrated that individualized rhythmic auditory stimulation aligned with a patient’s cadence can significantly improve gait performance, enjoyment, and therapy adherence (12, 13). Moreover, telerehabilitation approaches incorporating wearable systems have proven effective in improving balance and motor planning in PD patients (14). Despite technological challenges such as data privacy, environmental stability of sensors, and lack of standardization, these systems are emerging as vital tools in the shift toward data-driven, personalized neurorehabilitation. Integrating such objective tools provides insight into not only motor improvements but also user experience and long-term adherence. These findings form the foundation upon which the present study builds.

While numerous studies have explored the use of wearable devices to enhance rehabilitation, few have quantitatively assessed the outcomes of individual rehabilitation sessions. Therefore, in this study, we aimed to quantify individual rehabilitation sessions and explore optimal approaches tailored to patient characteristics.

## Methods

### Study design and participants

We enrolled patients diagnosed with PD, PSP, and CBS residing in an elderly care facility and wore a three-axis multisensory wristband-type wearable device (iAide^™^2, TOKAI; Gifu, Japan) from April to May 2022. The device recorded differences in body surface temperature, pulse rate before and after rehabilitation, calories consumed, steps taken, and activity index of upper and lower extremity (15). Inclusion criteria were: (1) aged ≥20 years at consent, (2) residents of an elderly care facility, (3) diagnosed with PD or atypical parkinsonism according to the diagnostic criteria (16–18), and (4) able to provide informed consent. Patients unable to wear the device and who had severe dementia (Mini-Mental State Examination (MMSE) score < 10) were excluded. The sample size was determined by participant availability during the recruitment period and not based on statistical power calculations. After obtaining consent, a functional training plan was developed by a trained functional trainer, and participants performed exercises based on the plan. Rehabilitation modalities included water stimulation bed, therapeutic massage, sit-to-stand training, gait training, cycle ergometer training, treadmill walking, stretching, balance training, calisthenics, and resistance training.

### Standard protocol approvals, registrations, and patient consent

This study was approved by the Juntendo University Ethics Committee (approval number: #M20-0294-M01). Written informed consent was obtained from all participants. This retrospective study analyzed data originally collected for clinical purposes.

### Study procedure

Rehabilitation was conducted in an elderly care facility where participants wore the iAide^™^2 on one upper and one lower limb. All participants were supervised by physical therapists to put on wearable devices. Participants continued their routine activities and received regular medications. Rehabilitation was performed 0–7 times per week and 0–3 times per day, depending on symptoms. Sessions were suspended temporarily during a COVID-19 outbreak. The water stimulation bed is a mechanical massage device that provides body stimulation via water pressure. Therapeutic massage was administered manually by a physical therapist to reduce muscle tension and improve motor symptoms (19). Sit-to-stand training involved repeated standing and sitting to strengthen lower limb muscles and improve mobility (20). Gait training was tailored to each participant to enhance walking stability (21). Cycle ergometer training and treadmill walking aimed to improve endurance and gait disturbances (22, 23). Stretching exercises, guided by a physical therapist, targeted limb and trunk flexibility. Balance training used tools like balance balls or one-legged stances to enhance stability. Calisthenics, performed under a physiotherapist’s guidance, improved endurance and flexibility (24). Resistance training utilized low to moderate loads to maintain and increase muscle strength (25). Exercises were performed for specified durations depending on participant needs.

### Data acquisition and analysis

Body surface temperature, pulse rate, activity index of upper and lower extremity, steps, and calories were recorded using iAide^™^2. Pulse rate, body surface temperature, activity index, steps, and calories were captured every minute. Temperature and pulse rate data were recorded from the upper limb device, and the difference between the start and end of each session was used to calculate. Activity index values were averaged from minute-by-minute data collected from the upper or lower limb during rehabilitation. Step counts were averaged from data recorded on the lower limb device. Calories were estimated from the activity index and participant weight, and average calorie consumption was displayed. The activity index was calculated using a proprietary algorithm corresponding to metabolic equivalents (METs) (26). Calories burned were computed as: 1.05 × METs × time (hr) × body weight (kg) (27). Data were log-transformed to a scale of 0–100 and visualized in radar charts, with each metric representing a chart axis.

### Statistical analysis

All statistical tests were performed using Python (version 3.12.0.). A *p*-value of <0.05 was considered statistically significant.

Before conducting any statistical tests, the normality of the data for each variable was assessed using the Shapiro–Wilk test. If the data did not follow a normal distribution, non-parametric tests were used for further analysis. Statistical analyses were performed to evaluate the differences between the pre-and post-intervention values and the change in scores for body surface temperature and pulse rate. Paired comparisons were conducted using the Wilcoxon signed-rank test. To account for the multiple comparisons performed, False Discovery Rate (FDR) correction was applied using the Benjamini-Hochberg procedure.

For the analysis of values across different rehabilitation groups, we used the Kruskal-Wallis test. For pairwise comparisons between groups, post-hoc Dunn’s test was performed. To account for multiple comparisons, *p*-values were corrected using the Benjamini-Hochberg procedure to control the FDR.

## Results

### Patient characteristics

A total of 49 patients with PD, PSP, and CBS participated in the study. The cohort comprised 19 males and 30 females, including 41 patients with PD, six with PSP, and two with CBS. The mean age at consent was 77.12 ± 7.64 years, disease duration was 10.67 ± 7.06 years, Hoehn and Yahr (HY) stage during “on” was 3.60 ± 0.89, and HY stage during “off” was 4.25 ± 0.81. The MMSE score was 19.28 ± 9.84, and the Functional Independence Measure (FIM) score was 64.23 ± 28.95. The total levodopa equivalent daily dose (LEDD) was 606.50 ± 284.51 (Table 1), calculated using standard conversion factors (28).

**Table 1:** Background and clinical features of the patients.

Characteristic	Baseline (*n* = 49)
Sex	Male = 19, Female = 30
Disease	PD = 41, PSP = 6, CBS = 2
Age at evaluation, y	77.12 ± 7.64 (54–91)
Disease duration, y	10.67 ± 7.06 (1–32)
H-Y (On state)	3.60 ± 0.89 (2–5)
H-Y (Off state)	4.25 ± 0.81 (2–5)
Total LEDD, mg	606.50 ± 284.51 (0–1358.80)
MMSE	19.28 ± 9.84 (0–30)
FIM	64.23 ± 28.95 (18–119)

Values are shown as mean ± SD (min-max) or n. PD, Parkinson’s disease; PSP, progressive supranuclear palsy; CBS, Corticobasal Syndrome; H-Y, Hoehn and Yahr scale; LEDD, Levodopa equivalent daily dose; MMSE, Mini-Mental State Examination; FIM, Functional Independence Measure.

### Quantification of each rehabilitation

Each rehabilitation session lasted 5–30 min and was administered as needed, depending on the patient’s symptoms. Individual patients occasionally underwent multiple sessions of the same rehabilitation type. Table 2 presents each rehabilitation modality and the total number of sessions administered. The number of sessions ranged from one session of water stimulation bed therapy to 52 sessions of resistance training, selected at the therapist’s discretion based on the patient’s condition. For each rehabilitation, we assessed the difference in body surface temperature before and after the session, calories expended, steps taken, change in pulse rate, and upper and lower extremity activity indices. The activity index was calculated in METs per minute using iAide^™^2.

**Table 2:** Characteristics of each rehabilitation.

Rehabilitation	Purpose	Duration (minute)	Times (total)
Water stimulation bed	Effects of water pressure massage on reducing muscle tension and improving flexibility.	10–15	1
Therapeutic massage	Manual massage to relieve muscle tension and improve motor symptoms.	5–10	45
Sit-to-stand training	Improvement of lower limb muscle strength through sit and standing movements.	5–10	5
Gait training	Improve gait and balance by performing walking movements in a manner that allows.	5–10	21
Cycle ergometer training	Bicycle pedaling exercise improves gait disturbance and endurance.	10–15	25
Treadmill	Maintaining and improving general endurance and acquiring walking rhythm.	5–15	10
Stretching	Improved flexibility of extremities and trunk muscles.	10–15	37
Balance training	Maintain balance in standing and sitting positions.	5–10	5
Calisthenics	Gymnastics improves general endurance and flexibility.	20–30	39
Resistance training	Strength training with low to moderate load to increase muscle strength.	5–10	52

Water stimulation bed, therapeutic massage, sit-to-stand training, gait training, cycle ergometer training, treadmill, stretching, balance training, calisthenics, resistance training were performed in this study. Duration indicates the actual time the rehabilitation was performed, and Times indicates the total number of times the rehabilitation was actually performed. Each rehabilitation session lasted 5–30 min, as needed, and was performed as many times as necessary, depending on the patient’s symptoms.

The body surface temperature was measured before and after each rehabilitation intervention. The water stimulation bed group increased from 31.5°C to 32.1°C, and the therapeutic massage group from 29.9 ± 2.7°C to 31.1 ± 2.4°C. The sit-to-stand training group increased from 29.0 ± 0.8°C to 30.2 ± 1.0°C, and the gait training group increased from 29.2 ± 1.9°C to 29.8 ± 1.6°C. The cycle ergometer training group rose from 29.9 ± 2.0°C to 30.2 ± 1.8°C, the treadmill group from 29.8 ± 1.9°C to 30.4 ± 1.5°C, and the stretching group from 29.5 ± 2.2°C to 30.1 ± 1.8°C. The balance training group increased from 30.6 ± 2.0°C to 30.9 ± 2.4°C. The calisthenics group slightly decreased from 30.5 ± 3.0°C to 30.4 ± 1.7°C, and the resistance training group decreased from 30.6 ± 1.8°C to 30.2 ± 1.7°C. No significant pre-post changes were observed within each group (Wilcoxon signed-rank test, Benjamini–Hochberg correction, *p* > 0.05). However, the stretching and treadmill groups showed significantly greater temperature increases compared to others (Kruskal-Wallis test, Benjamini–Hochberg correction, *p* < 0.05).

The pulse rate (PR) was measured before and after each rehabilitation intervention. In the water stimulation bed group, data for standard deviation were not available and therefore were excluded. In the therapeutic massage group, the PR decreased from 65.9 ± 14.7 to 64.8 ± 14.3 bpm. In the sit-to-stand training group, the PR increased from 62.0 ± 17.3 to 64.8 ± 8.3 bpm. In the gait training group, the PR increased slightly from 64.7 ± 13.6 to 67.0 ± 24.0 bpm. In the cycle ergometer training group, the PR decreased from 69.1 ± 14.1 to 63.6 ± 19.8 bpm. The treadmill group showed a notable increase from 62.0 ± 17.7 to 82.6 ± 49.7 bpm. In the stretching group, the PR slightly decreased from 63.2 ± 12.2 to 61.5 ± 12.8 bpm. In the balance training group, the PR decreased from 82.0 ± 31.6 to 66.2 ± 19.0 bpm. In the calisthenics group, the PR increased from 65.3 ± 7.9 to 68.9 ± 19.6 bpm. Lastly, in the resistance training group, the PR increased slightly from 68.5 ± 15.3 to 69.9 ± 15.1 bpm. No statistically significant differences were observed between pre-and post-intervention PR within each rehabilitation group (Wilcoxon signed-rank test with Benjamini-Hochberg correction, *p* > 0.05). Additionally, no significant differences in PR change were found between rehabilitation interventions (Kruskal-Wallis test, Benjamini-Hochberg correction, *p* > 0.05).

The activity index (AI) of the upper limb was 0.0 ± 0.0 in the water stimulation bed, 2.3 ± 7.9 in the therapeutic massage, 0.0 ± 0.0 in the sit-to-stand training, 3.6 ± 14.0 in the gait training, 15.8 ± 35.6 in the cycle ergometer, 30.4 ± 44.4 in the treadmill, 1.1 ± 6.6 in the stretching, 2.4 ± 7.6 in the balance training, 3.4 ± 12.2 in the calisthenics, and 1.0 ± 5.9 in the resistance training group. No significant differences were observed across groups (Kruskal-Wallis test, Benjamini-Hochberg correction, *p* > 0.05).

The AI of lower limb was 3.6 ± 14.0 in the gait training group, 1.0 ± 5.9 in the resistance training group, 0.0 ± 0.0 in the water stimulation bed group, 15.8 ± 35.6 in the cycle ergometer group, 1.1 ± 6.6 in the stretching group, 30.4 ± 44.4 in the treadmill group, 2.4 ± 7.6 in the balance training group, 2.3 ± 7.9 in the Therapeutic massage group, 0.0 ± 0.0 in the sit-to-stand training group, and 3.4 ± 12.2 in the calisthenics group. No significant differences were observed across groups (Kruskal-Wallis test, Benjamini-Hochberg correction, *p* > 0.05).

The step was 3.6 ± 14.0 in the gait training group, 1.0 ± 5.9 in the resistance training group, 0.0 ± 0.0 in the water stimulation bed group, 15.8 ± 35.6 in the cycle ergometer training group, 1.1 ± 6.6 in the stretching group, 30.4 ± 44.4 in the treadmill group, 2.4 ± 7.6 in the balance training group, 2.3 ± 7.9 in the Therapeutic massage group, 0.0 ± 0.0 in the sit-to-stand training group, and 3.4 ± 12.2 in the calisthenics group. No significant differences were observed across groups (Kruskal-Wallis test, Benjamini-Hochberg correction, *p* > 0.05).

The expended calories were 3.6 ± 14.0 in the gait training group, 1.0 ± 5.9 in the resistance training group, 0.0 ± 0.0 in the water bed group, 15.8 ± 35.6 in the cycle ergometer group, 1.1 ± 6.6 in the therapeutic massage group, 30.4 ± 44.4 in the treadmill group, 2.4 ± 7.6 in the balance training group, 2.3 ± 7.9 in the Therapeutic massage group, 0.0 ± 0.0 in the sit-to-stand training group, and 3.4 ± 12.2 in the calisthenics group. No significant differences were observed across groups (Kruskal-Wallis test, Benjamini-Hochberg correction, *p* > 0.05).

**Figure 1:**
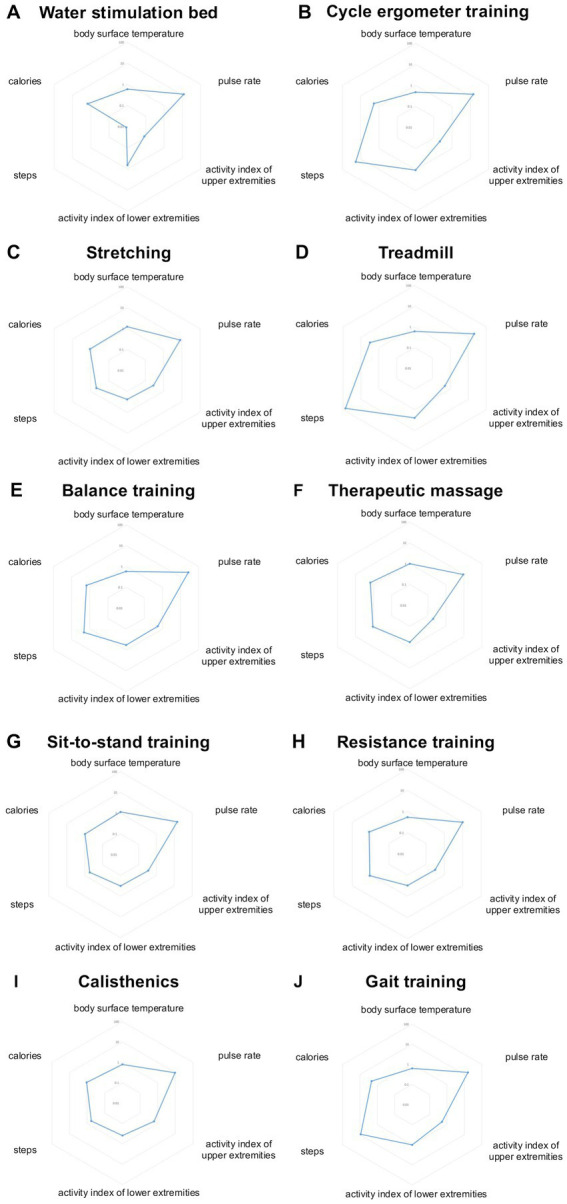
Quantification of rehabilitation. Illustrates the quantified metrics for each rehabilitation modality. The following physiological responses were evaluated: the difference in body surface temperature before and after rehabilitation (body temperature), the calories consumed during rehabilitation (calories), the number of steps taken during rehabilitation (steps), the difference in pulse rate before and after rehabilitation (pulse rate), and the activity index of the upper and lower extremities during rehabilitation (activity index). These metrics were evaluated for the following rehabilitation modalities: **(A)** water stimulation bed, **(B)** cycle ergometer training, **(C)** stretching, **(D)** treadmill, **(E)** balance training, **(F)** therapeutic massage, **(G)** sit-to-stand training, **(H)** resistance training, **(I)** calisthenics, and **(J)** gait training.

These results are summarized in Figure 1.

## Discussion

In the present study, we used wearable devices to record the motor and non-motor effects of various rehabilitation interventions for patients with PD and Parkinson’s syndrome. These interventions included water stimulation beds, therapeutic massage, sit-to-stand training, gait training, cycle ergometer training, treadmill use, stretching, balance training, calisthenics, and resistance training. We successfully quantified and presented, in graphical form, several rehabilitation-related metrics: body surface temperature differences, calories expended, number of steps taken, pulse rate changes before and after rehabilitation, and AI of upper and lower limbs, both during and surrounding rehabilitation sessions.

Rehabilitation is widely recognized as a critical treatment strategy for neurodegenerative disorders. It provides three principal benefits. First, it has symptomatic effects. The effectiveness of exercise therapy in alleviating symptoms has long been documented (29). For example, patients with PD who engage in exercise therapy demonstrate significantly reduced motor symptom scores compared to those who do not (9, 30). Moreover, exercise therapy may potentiate the effects of antiparkinsonian medications (31, 32), and it has been shown to enhance cognitive function and attention in patients (33). Second, rehabilitation may serve as a disease-modifying intervention. Maintaining high levels of physical activity has been linked to a more favorable clinical course in patients with PD (34–36). Animal studies have shown that the loss of dopamine-producing cells is greatly suppressed in the group treated with exercise therapy in PD model mice. This effect is thought to stem from improved mitochondrial function and elevated levels of brain-derived and glial cell line–derived neurotrophic factors (37). Third, rehabilitation may act as a preventive measure. Regular, intense physical activity in middle age has been associated with a reduced risk of developing PD and Parkinson’s syndrome (38–43).

Current exercise recommendations often lack specific guidance regarding frequency, intensity, duration, or necessary adjustments based on patient symptoms. The WHO advises older adults (aged ≥65 years) to engage in either 150 min of moderate-intensity aerobic exercise or at least 75 min of vigorous-intensity exercise per week (44). In addition to intensity metrics such as METs, recent recommendations also consider heart rate targets. Exercise performed at 60–80% of heart rate reserve or 70–85% of maximum heart rate is considered optimal (8). However, these benchmarks for healthy individuals may not be suitable for all the patient with PD. Increased physical activity typically results in elevated body temperature and heart rate, especially during dynamic exercise, which activates the sympathetic nervous system more than static movements (45). Nonetheless, in our findings, passive interventions such as stretching and hand massage produced more pronounced increases in body surface temperature compared to treadmill and ergometer use, despite significantly lower step counts and activity levels. This may reflect limitations in mobility due to individual patient characteristics and the presence of autonomic neuropathy in PD. Therefore, it is important to establish individualized exercise targets tailored to each patient’s condition.

Moderate-intensity progressive resistance training, performed 2–3 times per week over 8–10 weeks, has been shown to significantly improve strength, balance, and motor symptoms in patients with early to moderate stages of PD (46). However, rehabilitation programs often incorporate complex elements, such as combined aerobic and strength training, as observed in the present study. In actual clinical practice, multiple modalities are frequently selected and combined from various available rehabilitation interventions. Although systematic reviews have attempted to evaluate the overall effects of physical therapy in PD, rather than focusing on isolated interventions, formal comparisons remain difficult. This is largely due to small sample sizes, methodological limitations, potential publication bias, and the diversity of exercise protocols employed (47). Additionally, the inherent difficulty of blinding exercise interventions may limit the availability of high-quality controlled trials. Therefore, understanding the characteristics and physiological profiles of each structured rehabilitation modality is an essential first step. It is also important to recognize that the same activity may produce different physiological responses depending on the situation. For instance, our findings showed that although treadmill walking resulted in a greater number of steps, more pronounced lower extremity movement, and elevated heart rate compared to standard walking training, it was associated with reduced upper extremity movement. While training quantification has been studied in sports medicine, few studies have attempted to quantify rehabilitation activity in neurological disorders (48). Therefore, we propose using wearable devices to quantify rehabilitation interventions through multiple physiological biomarkers. Wearable technologies hold promises for assessing both exercise and symptom profiles in PD (49, 50). Quantification enables the development of personalized rehabilitation programs and facilitates evaluation of intervention effectiveness. Furthermore, such technology could support independent rehabilitation in the absence of a therapist, and serve as a foundation for future high-quality research, including blinded studies.

One limitation of the present study is the lack of standardization in rehabilitation protocols. Ideally, methods should be standardized with respect to aerobic exercise intensity (e.g., based on heart rate), resistance load, balance training techniques, and use of equipment such as water stimulation beds. Rehabilitation modalities were administered based on therapist discretion, reflecting real-world conditions but resulting in heterogeneous exposure and limited comparability. Another limitation is the lack of control group consisting of either healthy participants or individuals undergoing rehabilitation without wearable monitoring. This was due to the real-world setting in an elderly care facility and ethical constraints. However, we acknowledge that the absence of a comparator limits causal inferences. Moreover, the sample size was determined by participant availability rather than statistical power calculation, which may reduce the ability to detect subtle effects. Furthermore, the mean MMSE score indicated moderate cognitive impairment, and participants with lower cognitive function may have had difficulty fully engaging with the rehabilitation sessions or following instructions, potentially affecting adherence and reducing the reliability of wearable-derived metrics. Future studies should consider stratifying by cognitive status or excluding patients with severe cognitive impairment to improve interpretability and data quality. Lastly, while our aim was to reflect the diversity of real-world parkinsonian syndromes, inclusion of patients with PSP and CBS introduced clinical heterogeneity that may have affected responsiveness to intervention, and the generalizability of the findings specifically to PD populations is limited. Our results should be interpreted as a proof-of-concept for the feasibility of rehabilitation quantification using wearable sensors, rather than as disease-specific efficacy data.

This investigation represents an initial step toward individualized rehabilitation in PD. Quantifying the physiological effects of each intervention enables informed combinations tailored to each patient’s needs. Using wearable devices, we successfully quantified discrete rehabilitation activities. These findings may help guide the development of personalized rehabilitation strategies aimed at optimizing functional outcomes in patients with PD.

## Data Availability

The raw data supporting the conclusions of this article will be made available by the authors, without undue reservation.
